# Satellite RNAs interfere with the function of viral RNA silencing suppressors

**DOI:** 10.3389/fpls.2015.00281

**Published:** 2015-04-24

**Authors:** Wan-Xia Shen, Phil Chi Khang Au, Bu-Jun Shi, Neil A. Smith, Elizabeth S. Dennis, Hui-Shan Guo, Chang-Yong Zhou, Ming-Bo Wang

**Affiliations:** ^1^National Citrus Engineering Research Center, Citrus Research Institute, Southwest UniversityChongqing, China; ^2^Commonwealth Scientific and Industrial Research Organisation Plant IndustryCanberra, ACT, Australia; ^3^Department of Plant Science, Waite Institute, Adelaide UniversityGlen Osmond, SA, Australia; ^4^State Key Laboratory of Plant Genomics, Institute of Microbiology, Chinese Academy of SciencesBeijing, China

**Keywords:** virus-encoded suppressor, satellite RNA, symptom attenuation, RNA silencing, microRNA expression

## Abstract

Viral satellite RNAs (satRNAs) are small subviral RNAs and depend on the helper virus for replication and spread. satRNAs can attenuate helper virus-induced symptoms, the mechanism of which remains unclear. Here, we show that two virus-encoded suppressors of RNA silencing (VSRs), *Cucumber mosaic virus* (CMV) 2b and *Tombusvirus* P19, suppress hairpin RNA (hpRNA)-induced silencing of a *β-glucuronidase* (GUS) gene in *Nicotiana benthamiana*. This suppression can be overcome by CMV Y-satellite RNA (Y-Sat) via the Y-Sat-derived small interfering RNAs (siRNAs), which bind to the VSRs and displace the bound hpGUS-derived siRNAs. We also show that microRNA target gene expression in *N. tabacum* was elevated by CMV infection, presumably due to function of the 2b VSR, but this upregulation of microRNA target genes was reversed in the presence of Y-Sat. These results suggest that satRNA infection minimizes the effect of VSRs on host siRNA and microRNA-directed silencing. Our results suggest that the high abundance of satRNA-derived siRNAs contributes to symptom attenuation by binding helper virus-encoded VSRs, minimizing the capacity of the VSRs to bind host siRNA and miRNA and interfere with their function.

## Introduction

SatRNAs (satRNAs) depend on their associated viruses (helper viruses) for replication, encapsidation, systemic movement and transmission, and are regarded as parasites of their helper viruses. They have small RNA genomes sized from 220 to 1400 nucleotides (nt), which share little or no sequence homology with the genome of helper viruses (Roossinck et al., 1992; Simon et al., 2004). The relatively large (800–1400 nt) satRNAs can encode one or a few proteins, but the majority of satRNAs, with <700 nt genome, possess no mRNA activity and do not encode any protein (Roossinck et al., 1992; Simon et al., 2004). Some satRNAs can induce specific symptoms in virus-infected plants (Garcia-Arenal and Palukaitis, 1999; Hu et al., 2009), for example, yellowing symptoms in *Nicotiana* species induced by Y satRNA (Y-Sat) of *Cucumber mosaic virus* (CMV) (Masuta and Takanami, 1989). Recent studies showed that the yellowing symptoms are the result of the silencing of the chlorophyll biosynthetic gene *CHLI* induced by Y-Sat-derived small interfering RNAs (siRNAs), which have a 22-nt sequence homologous to *CHLI* (Shimura et al., 2011; Smith et al., 2011). In contrast to Y-Sat, most satRNAs do not induce their own symptoms. Another feature of satRNAs is the attenuation of helper virus-induced symptoms (Roossinck et al., 1992; Simon et al., 2004). This property has been used in transgenic plants for viral disease prevention (Baulcombe et al., 1986; Gerlach et al., 1987; Harrison et al., 1987). However, how satRNAs attenuate the symptoms remains a matter for debate. It was proposed that satRNAs might compete with the helper viruses for RNA replicase or increase antiviral silencing against the accumulation of the helper viruses (Piazzolla et al., 1982; Wu and Kaper, 1995; Hou et al., 2011). However, in some cases, satRNAs attenuate the symptoms but do not reduce the accumulation of helper viruses (Harrison et al., 1987; Moriones et al., 1992). Therefore, other unknown factors may also be involved in the regulation of helper virus-induced symptoms.

RNA silencing is a sequence-specific RNA degradation process induced by 21–24-nt small RNAs (sRNAs) processed from double-stranded RNA (dsRNA) or self-complementary hairpin RNA (hpRNA) by Dicer or Dicer-like protein, an RNase III-like endoribonuclease. One strand of each sRNA is loaded onto the RNA-induced silencing complex (RISC) and guides Argonaute (AGO) proteins in the RISC to cleave targeted RNA (Zamore, 2001). In plants RNA silencing has been recognized as an anti-viral defense mechanism by which siRNAs derived from the virus guide the degradation of the viral RNA in the infected plant (Baulcombe and Molnar, 2004; Ruiz-Ferrer and Voinnet, 2009). To overcome this host defense mechanism, viruses have evolved a counter-defense strategy by encoding suppressors of RNA silencing (VSRs) (Zhang et al., 2006; Shimura and Pantaleo, 2011; Pumplin and Voinnet, 2013). A predominant action mode of VSRs is to bind the siRNAs preventing their entry into the RISC and degradation of the viral RNA (Lakatos et al., 2006). Beside viral siRNAs, VSRs also interfere with the function of host small RNAs including microRNAs (miRNAs) (Kasschau et al., 2003; Chapman et al., 2004; Zhang et al., 2006; Lewsey et al., 2007). miRNAs play a critical role in processes such as cell division, leaf formation, and flower development in plants (Voinnet, 2009). Therefore, while VSRs help the viruses to evade antiviral RNA silencing by the host, they can also induce symptoms by disrupting functions of specific miRNA species, which regulate host plant development (Zhang et al., 2006; Lewsey et al., 2007; Jay et al., 2011; Du et al., 2014).

In this study, we used an Agrobacterium infiltration (agro-infiltration)-based transient expression system to investigate how the satRNA of CMV Y (Y-Sat) affected the ability of CMV-encoded VSR 2b and *Tombusvirus*-encoded VSR P19 to suppress hpRNA-induced gene silencing in *Nicotiana benthamiana*. Both VSRs suppressed hpRNA-induced gene silencing, but in the presence of Y-Sat, this VSR effect was reversed. Using RNA immunoprecipitation, we showed that this reversion is due to the bound hpRNA-derived siRNAs being displaced from the VSRs by Y-Sat-derived siRNAs. We also found that Y-Sat infection minimizes the over-expression of target genes of endogenous miRNAs caused by CMV infection, indicating that Y-Sat inhibited the effect of the 2b VSR on the function of host miRNAs.

## Materials and methods

### Plant growth, virus inoculation, plasmid constructs, and plant transformation

*N. benthamiana* and *N. tabacum* (HcPro transgenic; Wang et al., 2004) plants were grown at 25°C under a 16-h light/8-h dark cycle. For virus infection, leaves of 3–5 week old plants were dusted with carborundum and rub-inoculated with leaf extracts of previously infected plants in 0.1 M phosphate buffer (pH 7.2).

The constructs *of GFP* and *P19* driven by the CaMV 35S promoter were previously described (Wood et al., 2009). The *2b* (wild-type) and *2bm* (mutant; created by substituting “C” for “U” in the start codon AUG, as well as three other AUG codons present in the 2b coding sequence) constructs were also previously described (Hou et al., 2011). The *GUS* construct (pIG121Hm) contains an intron in the coding sequence, which was designed to prevent GUS expression in bacterial cells (Ohta et al., 1990; Ishikawa, 2009). The *hpGUS* construct was previously reported, designed to express a long GUS hairpin RNA with a 563 bp dsRNA arm and an 1113 nt loop (Wang and Waterhouse, 2000). All of the above constructs were introduced into *A. tumefaciens* strains AGL1 (*GFP* and *hpGUS*) or GV3101 (pIG121Hm, *2b*, *2bm*, and *P19*).

### *Agrobacterium* infiltration assay

*A. tumefaciens* strains containing plant expression constructs were grown overnight at 28°C in Luria-Bertani medium (LB) containing appropriate antibiotics. After centrifugation, Agrobacterium cells were re-suspended in buffer containing 10 mM MgCl_2_ and 150 μg/mL acetosyringone, with a final optical density at 600 nm (OD_600_) of 0.7. The bacterial suspensions were incubated at room temperature for ~3 h and then infiltrated into expanded *N. benthamiana* leaves using flat-pointed syringe (Liu and Lomonossoff, 2002). For protein and RNA isolation, agro-infiltrated leaf sections were visualized under a blue light torch (NightSea™, DFP-1™, for exciting green light emission), excised with a pair of scissors and immediately frozen in liquid nitrogen at 4 or 5 days post agro-infiltration (dpa).

### MUG assay of GUS expression

For fluorometric MUG (4-methyl-umbelliferyl-β-D-gulcuronide) assay, agro-infiltrated leaf sections were homogenized in extraction buffer (50 mM sodium phosphate, 10 mM Na_2_EDTA, 0.1% sarcosyl, 10 mM β-mercaptoehtanol, pH 7.0). The homogenates were subjected to centrifugation for 5 min at 4°C at 16,000 × g, and the supernatants were collected and kept on ice. Concentration of total protein was determined using the Bradford reagent. MUG reactions were carried out at 37°C and GUS activities were calculated essentially as previously described (Chen et al., 2005) using a fluorescence plate reader (Wallac 1420 VICTOR, PerkinElmer, Turku, Finland).

### RNA isolation, northern blot analysis, and RT-PCR

Total RNA was isolated from *N. benthamiana* and *N. tabacum* leaves or leaf sections using Trizol reagent (Invitrogen) following the manufacturer's instructions. Isopropanol precipitation of the total RNA was carried out overnight at −20°C to maximize recovery of the small RNA fraction.

For detection of large RNAs in northern blot hybridization, total RNA (~5–10 μg) was separated in formaldehyde-agarose gel (Sambrook et al., 1989) and blotted onto Hybond-N membrane (Amersham Life Science, NSW, Australia) in 10× SSC. The membrane was hybridized with [α−^32^P] UTP-labeled RNA probes obtained by *in vitro* transcription using radioactive UTP from the respective sequences (Supplemental Text 1) cloned into pGEM plasmids (Promega, NSW, Australia). Hybridization was performed as previously described (Wang et al., 2001).

For detection of small RNAs in northern blot hybridization, approximately 10–20 μg of total RNA were separated in a denaturing (urea) 17% polyacrylamide gel. RNA was electro-blotted onto a Hybond-N^+^ membrane (Amersham Life Science) in 0.5× TBE buffer, UV cross-linked, and hybridized overnight at 42°C with [α−^32^P] UTP labeled, carbonate buffer-fragmented RNA probe (Wang et al., 2001). The hybridized membrane was washed three times (20 min per wash) at 40°C with 2× SSC containing 0.2% SDS. Both large and small RNA northern blots were visualized on a phosphoimager (Fujifilm FLA 5000, Tokyo, Japan).

For RT-PCR or qRT-PCR analysis of 2b, HcPro mRNA, or miRNA target genes, total RNA (~1 μg) isolated from infected and uninfected leaves was reverse-transcribed with Superscript III reverse transcriptase (Invitrogen) following the manufacturer's instruction using oligo-dT_23_ as primer. The synthesized cDNA was amplified using 2b, HcPro or miRNA target gene-specific primers (see Supplemental Text 2 for primer sequences) with 56°C (2b) or 60°C (HcPro and miRNA target genes; qRT-PCR) as the annealing temperature. PCR cycle numbers (2b) are given on the gel pictures. Real-time PCR was performed in the Rotor-Gene 6000 (Corbett Life Science, San Francisco, USA) real-time rotary analyser using SYBR Green reagent and Platinum Taq polymerase (Invitrogen) in three technical replicates for each sample. mRNA encoding the *N. tabacum* elongation factor 1α (EF1α) was amplified for use as loading control.

### RNA immunoprecipitation

RNA immunoprecipitation was performed as described by Omarov et al. (2006) with minor changes. Briefly, tissues (2 g) were ground into fine powder in liquid nitrogen and re-suspended in 2 mL ice-cold extraction buffer with 150 mM HEPES (pH 7.5), 200 mM NaCl, 1 mM EDTA, 2 mM DTT, 16 unit RNaseOUT (Invitrogen, Victoria, Australia) and 20 μL Plant protease inhibitors (Sigma-Aldrich, NSW, Australia). The homogenized plant material was filtered through two layers of miracloth (Calbiochem, Victoria, Australia) and centrifuged twice at 10,000 × g at 4°C for 15 min. The supernatant was pre-cleared in 10 μL Salmon Sperm DNA/Protein G agarose beads (Millipore, Victoria, Australia) for 30 min at 4°C under constant rotation. An aliquot (800 μL) of pre-cleared lysate was then immunoprecipitated with 2 μL of P19 rabbit antibodies (A gift from Prof. Herman Bertus Scholthof, Texas A&M University) and 30 μL of Salmon Sperm DNA/Protein G agarose beads (Millipore) for 3 h at 4°C under constant rotation. Following immunoprecipitation, the agarose-antibody-P19 /RNA complexes were washed six times in extraction buffer. P19/RNA complexes were eluted with 100 μL of RNA-IP elution buffer [100 mMTris-HCl pH 8.0, 10 mM EDTA, 1% SDS, 40 units of RNaseOUT (Invitrogen), 20 μg of Proteinase K (Sigma-Aldrich)] at 65°C for 1 h. RNA was purified by extraction with an equal volume of acidic phenol/chloroform (Ambion, Applied Biosystems, Victoria, Australia), followed by overnight ethanol precipitation in the presence of acidic sodium acetate and 20 μg glycogen (Fermentas, Thermo fisher scientific, Victoria, Australia) at −80°C, and resuspended in DEPC-treated H_2_O for small RNA northern blot hybridization. For Western blot analysis of the pull-down material, elution step was omitted and washed beads were stored at −20°C for Western blotting.

### Western blot analysis

Beads containing the immunoprecipitated P19/RNA complexes were denatured at 95°C for 5 min with addition of Western blot loading buffer (Bromophenol blue, 0.125 M Tris-HCl, 4% SDS, 20% glycerol, 2% β-mercaptoethanol, pH 6.8). The proteins were separated on a 15% SDS polyacrylamide gel, transferred to Immobilon P membrane (Millipore) and detected by chemiluminescence using 1/2500 anti-P19 antibody (Omarov et al., 2006) and 1/5000 anti-rabbit Ig HRP conjugate (Chemicon, Victoria, Australia).

## Results

### Y-Sat reduces the symptoms caused by the helper virus Fny-CMV

**Figure 1:**
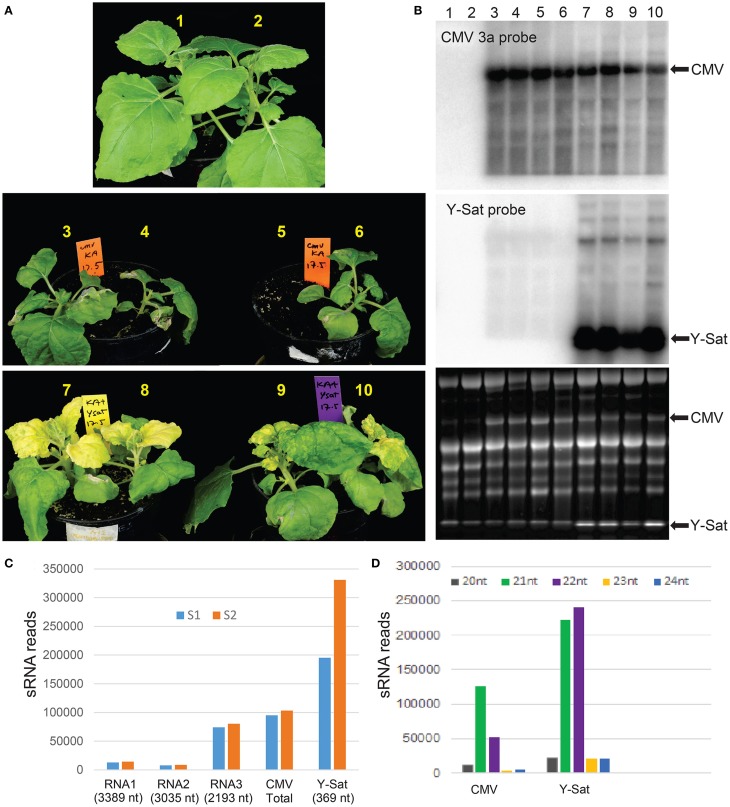
**Y-Sat infection reduces the severity of CMV-caused symptoms and is associated with high abundance of Y-Sat siRNAs. (A)** *N. benthamiana* plants co-infected with both Fny-CMV and Y-Sat (bottom) (12 days post inoculation) are healthier and bigger than those infected with Fny-CMV alone (middle), despite the Y-Sat-specific bright yellowing symptoms. Note that plant #5 was very weak and accidentally uprooted prior to photographing. **(B)** Northern blot analysis of plants in **(A)**, showing high amounts of Fny-CMV RNA in both CMV and CMV+Y-Sat-infected plants, although CMV RNA abundance is in general reduced in Y-Sat-infected plants. The CMV RNA3 and Y-Sat genomic RNA bands are indicated in both the hybridized northern blot (top and middle) and the stained RNA gel (bottom). **(C)** Total number of 20–24 nt sRNA reads perfectly mapped to Q-CMV (RNA1, RNA2, RNA3, and whole genome) and Y-Sat in two Q-CMV+Y-Sat-infected *N. tabacum* samples (S1 and S2). **(D)** Size distribution of Q-CMV and Y-Sat-derived sRNAs.

The strains of *Cucumber mosaic virus* (CMV) are classified into two subgroups, I and II, based on their serology, symptomatology and sequence homology (Palukaitis et al., 1992). Fny-CMV is a subgroup I strain and induces severe symptoms in many host plant species (Zhang et al., 1994). Its encoded VSR 2b has been shown to be a strong virulence contributor (Shi et al., 2002). In *N. benthamiana*, Fny-CMV not only induced severe symptoms, but also constrained plant growth (Figure 1A). However, in the presence of Y-Sat, the severity of the Fny-CMV-caused symptoms was reduced and plant growth improved, despite the bright yellow symptoms typical of Y-Sat infection (Figure 1A). This demonstrates that Y-Sat attenuates the symptoms induced by Fny-CMV, which is consistent with previous reports (Hou et al., 2011). Northern blot hybridization showed that Y-Sat co-infection reduced the level of CMV RNA, but not dramatically (Figure 1B).

### Y-Sat is the dominant source of viral siRNAs in infected plants

Previous studies by northern blotting and small-scale sequencing showed that the replication of Y-Sat is accompanied by high levels of sRNAs derived from Y-Sat (Wang et al., 2004; Ebhardt and Unrau, 2009). To further demonstrate this, we obtained two sets of sRNA sequence data from *N. tabacum* plants co-infected with Q-CMV (a subgroup II CMV) and Y-Sat using Illumina deep sequencing technology, and analyzed the relative abundance of Y-Sat and CMV-derived sRNAs. A total of 195,291 and 330,965 reads of 20–24 nt sRNAs (from a total of approximately 8 million sRNA reads each) were perfectly mapped to the Y-Sat genome in the two samples S1 and S2, respectively (Figure 1C). These numbers are approximately 2 and 3 times as much as the total 20–24 nt sRNA reads perfectly mapped to the Q-CMV genome (95,073 and 103,443 for S1 and S2, respectively) (Figure 1C). This is despite the 23 fold bigger size of the CMV genome (8617 nt) than the Y-Sat genome (369 nt), indicating that the Y-Sat genome has approximately 50 fold more sRNA coverage than the CMV genome, which is consistent with the previously reported small-scale sequencing result (Ebhardt and Unrau, 2009). This relatively high abundance of Y-Sat sRNAs could result from a relatively high proportion of Y-Sat RNA in the total viral RNA population of the Q-CMV+Y-Sat-infected *N. tabacum* plants, but a previous report showed that Y-Sat RNA represents about 31–38% of the total viral RNAs (Takanami, 1981), well below the 2~3 fold level of Y-Sat sRNAs.

### Y-Sat RNA interferes with the function of 2b in *Nicotiana benthamiana* leaves

Cucumoviral 2b proteins are essential for cucumoviruses to cause viral symptoms (Ding et al., 1996; Shi et al., 2002; Soards et al., 2002; Zhang et al., 2006; Lewsey et al., 2007), while Y-Sat infection reduces the symptoms (Figure 1). This led us to investigate if Y-Sat infection interferes with the 2b functions. We developed an Agrobacterium infiltration (agro-infiltration)-based transient expression system. In this system equal amounts of VSR-expressing constructs were agro-infiltrated into *N. benthamiana* leaves with infection of Q-CMV or Q-CMV plus Y-Sat, and mock-treated plant leaves, and the level of hpRNA-induced silencing of the β-glucuronidase (GUS) reporter gene in the leaves was measured. We used the subgroup II CMV strain, Q-CMV, as the helper virus to infect *N. benthamiana* instead of Fny-CMV. This was because Q-CMV encodes a relatively weak 2b VSR (Chapman et al., 2004; Zhang et al., 2006; Ye et al., 2009) and the level of VSR activity from the infecting Q-CMV is negligible compared to that from the agro-infiltrated VSR constructs expressing a strong VSR (SD-CMV 2b and tombusvirus P19) at relatively high levels. This allowed us to examine the effect of Y-Sat specifically on the agro-infiltrated VSR. In addition, Q-CMV only induces mild symptoms in *N. benthamiana* and allowed the normal leaf expansion required for agro-infiltration. Furthermore, Q-CMV, like Fny-CMV, supports high levels of Y-Sat replication and Y-Sat siRNA accumulation (Figure 1C and Wang et al., 2004). The following constructs were used in the agro-infiltration assay: GUS reporter gene (*GUS*), GUS silencing-inducing hpRNA (*hpGUS*), strong 2b (*2b*) VSR encoded by the subgroup I Shan-Dong (SD)-CMV (Hou et al., 2011), and green florescent protein gene (*GFP*) as a visual marker. A construct expressing a mutant 2b gene (*2bm*), in which the start codon and three other in-frame AUG codons of the 2b coding sequence were mutated (Duan et al., 2012), was used as a negative control. Gene expression in all of these constructs was driven by a constitutive *Cauliflower mosaic virus* (CaMV) 35S promoter.

**Figure 2:**
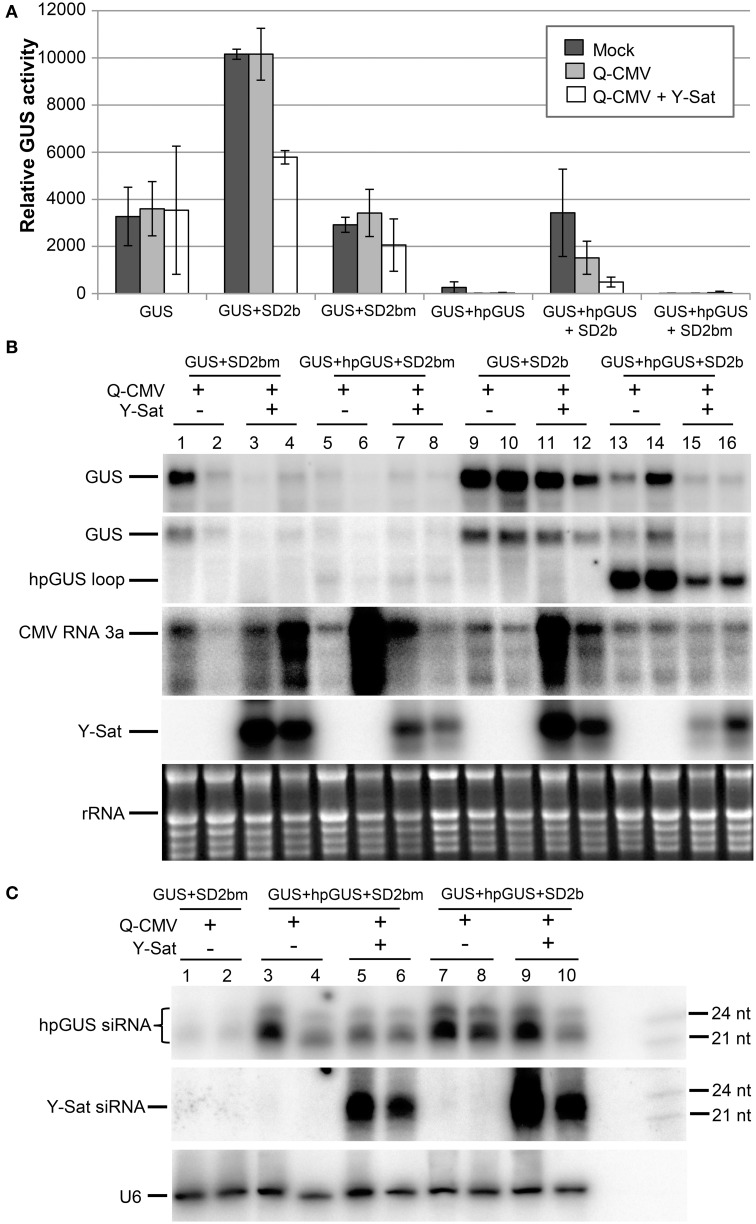
**The suppression of hpRNA-induced GUS silencing by agro-infiltrated 2b is released by CMV+Y-Sat infection**. **(A)** MUG assay of GUS expression in Mock (inoculated only with buffer), Q-CMV and Q-CMV+Y-Sat-infected *N. benthamiana* leaves infiltrated with the constructs indicated. The plants were infiltrated at 11 or 13 days after inoculation. The GUS activity was analyzed at 4 days post agroinfiltration (dpa). **(B)** Northern blot hybridization analysis of *GUS* expression in a subset of the samples described in **(A)**. Ten microgram of total RNA from each sample was hybridized with an antisense GUS probe to detect *GUS* mRNA (top panel), a probe complementary to the loop of the hpGUS RNA to detect both *GUS* mRNA and *hpGUS* loop fragments (second panel), a CMV RNA 3a probe to detect CMV (third panel), and a Y-Sat probe to detect Y-Sat (forth panel) in the agroinfiltrated tissues. Ethidium bromide-stained rRNA bands are used as a loading control (bottom panel). **(C)** Northern blot hybridization analysis of small RNAs in a subset of the samples described in **(A)**. Twenty microgram of total RNA each was hybridized with a RNA probe corresponding to the dsRNA stem region of hpGUS to detect hpGUS-derived siRNA (top panel), an antisense Y-Sat RNA probe to detect Y-Sat-derived siRNAs (middle panel) and a U6 probe to show loading control (bottom panel). The 21 and 24-nt sRNA size markers are loaded in the last lane on the right. As can be seen, the level of *hpGUS*-derived siRNAs is not significantly affected by Q-CMV Y-Sat infection. SD2b or SD2bm, wild-type or mutant 2b derived from the group I Shandong (SD) strain of CMV.

As shown by the fluorometric MUG (4-methylumbelliferyl-β-D-glucuronide) assay in Figure 2A, without *2b* co-infiltration, strong GUS silencing was induced by *hpGUS* in all uninfected (mock), Q-CMV-infected (Q-CMV), and Q-CMV+Y-Sat co-infected plants. Northern blotting confirmed that the *GUS* gene was silenced at the mRNA level (Figure 2B, top two panels). When *GUS* and *hpGUS* were co-infiltrated with *2bm*, *GUS* silencing was not suppressed (Figures 2A,B). However, when *GUS* and *hpGUS* were co-infiltrated with *2b*, the *GUS* activity was recovered substantially in the mock and Q-CMV-infected plants, but this was not the case for Q-CMV+Y-Sat co-infected plants, which continued to show strong GUS silencing (Figures 2A,B). These results indicate that 2b possesses the ability to suppress the hpRNA-induced GUS silencing but this ability is greatly inhibited in the presence of Y-Sat. There were variations in CMV RNA3 levels among the different samples, with a few samples containing particularly high amounts of CMV RNA3, although the four *GUS*+*hpGUS*+*2b*-infiltrated samples showed relatively uniform levels (Figure 2B). The variations could result from different age or infection state of the leaf tissues harvested because they did not seem to be due to the presence or absence of Y-Sat. CMV-infected samples did not show enhanced GUS expression in comparison to mock-treated ones (Figure 2A), and the variations in CMV RNA3 level did not have a clear effect on GUS expression levels (Figure 2B), presumably because of the weak VSR activity of the Q-CMV 2b.

Northern blot analysis of sRNAs corresponding to the 563-bp dsRNA arm region of the *hpGUS* transcript (Wang and Waterhouse, 2000) showed that Y-Sat did not increase the accumulation of *hpGUS*-derived siRNAs in the absence (Figure 2C, lanes 3–6) or presence (lanes 7–10) of *2b*. This indicates that the enhanced GUS silencing upon Y-Sat infection was not caused by an increase in the amount of *hpGUS*-derived siRNAs. In addition, a semi-quantitative RT-PCR analysis showed that the expression level of the agro-infiltrated *2b* gene was not affected by Q-CMV or Q-CMV+Y-Sat infection (Supplemental Figure S1). These results indicate that Y-Sat minimized the suppression of the hpRNA-induced GUS silencing mediated by 2b.

Surprisingly, when a probe complementary to the loop of the hpGUS RNA was used, an intense band of the predicted loop size (~1.1 kb) was detected in the *2b*-infiltrated tissues (Figure 2B, 2nd panel; lanes 13–16), indicating that the 2b protein enhanced the accumulation of the hpRNA loop. Loop fragments have been known to accumulate in hpRNA-expressing plants (Wang et al., 2008). However, the intensity of the hybridized loop fragment from the tissues infected with Q-CMV+Y-Sat was much weaker than that from the tissues infected with Q-CMV alone (lanes 15–16 vs. lanes 13–14). This further suggests that Y-Sat interferes with 2b function.

### Y-Sat RNA interferes with VSR P19 function in *N. benthamiana* leaves

**Figure 3:**
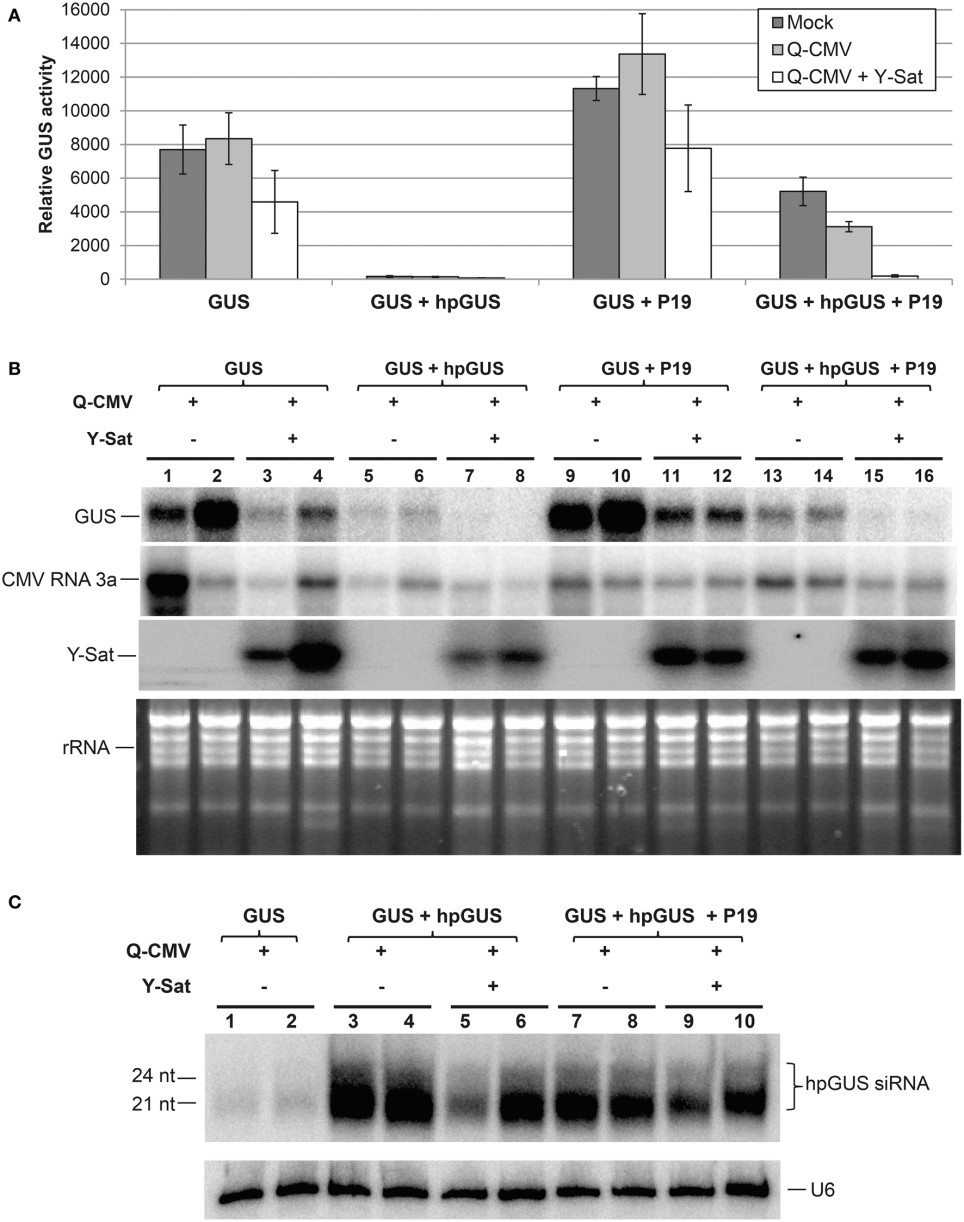
**The suppression of hpRNA-induced GUS silencing by agro-infiltrated P19 is released by CMV+Y-Sat infection**. **(A)** MUG assay of GUS expression in Mock, Q-CMV and Q-CMV+Y-Sat-infected (17 days post incoculation) *N. benthmaiana* leaves infiltrated with the indicated constructs at 5 dpa. **(B)** Northern blot hybridization analysis of *GUS* expression in a subset of the samples described in **(A)**. Five microgram of total RNA was hybridized with an antisense GUS probe to detect *GUS* mRNA levels (top panel), a CMV RNA 3a probe to measure CMV levels (second panel) and a Y-Sat probe to measure Y-Sat levels (third panel) in the agroinfiltrated tissues. Ethidium bromide-stained rRNA bands were used as loading control (bottom panel). **(C)** Northern blot hybridization analysis of small RNAs in a subset of the samples described in **(A)**. Ten microgram of total RNA each was hybridized with a RNA probe corresponding to the dsRNA stem region of hpGUS to detect hpGUS-derived siRNA (top panel) and a U6 probe to show loading control (bottom panel). The sizes of the detected siRNAs are indicated. As can be seen, the level of *hpGUS*-derived siRNAs is not significantly affected by Q-CMV Y-Sat infection.

The tombusvirus-encoded P19 is a well-characterized VSR that suppresses RNA silencing via binding and sequestering sRNAs (Vargason et al., 2003; Baulcombe and Molnar, 2004; Lakatos et al., 2006). We examined if Y-Sat also interferes with P19 functions. The same agro-infiltration system used above for 2b was applied. P19 suppressed *hpGUS*-induced GUS silencing in mock and Q-CMV-infected *N. benthaminana* tissues, and the suppression was negated in Q-CMV+Y-Sat-infected tissues (Figures 3A,B). In addition, the presence of Y-Sat did not increase the accumulation of *hpGUS*-derived siRNAs (Figure 3C), indicating that the recovery of GUS silencing in Q-CMV+Y-Sat-infected tissues was not due to an increase in the amount of *hpGUS*-derived siRNAs. These results indicate that Y-Sat enhances hpRNA-induced silencing in the agro-infiltrated *N. benthamiana* leaves by interfering with the function of P19 minimizing P19-mediated suppression of silencing. Thus, Y-Sat may use the same mechanism to interfere with the functions of 2b and P19.

It is worth mentioning that both 2b and P19 enhanced GUS expression in the *GUS*-infiltrated plants lacking *hpGUS* (Figures 2A,B and Figures 3A,B; compare “GUS” or “GUS+2bm” with “GUS+2b” or “GUS+P19”). This probably resulted from VSR-mediated suppression of sense co-suppression or posttranscriptional gene silencing that occurs to agro-infiltrated transgenes in *N. benthamiana* leaves (Voinnet et al., 1999). In the presence of Y-Sat, part of the enhancement of GUS expression by 2b or P19 was lost (Figures 2B, 3B; compare lanes 11–12 with lanes 9–10), further supporting the conclusion that Y-Sat interferes with the function of 2b and P19.

### Y-Sat infection minimizes the effect of CMV infection on expression of miRNA target genes

**Figure 4:**
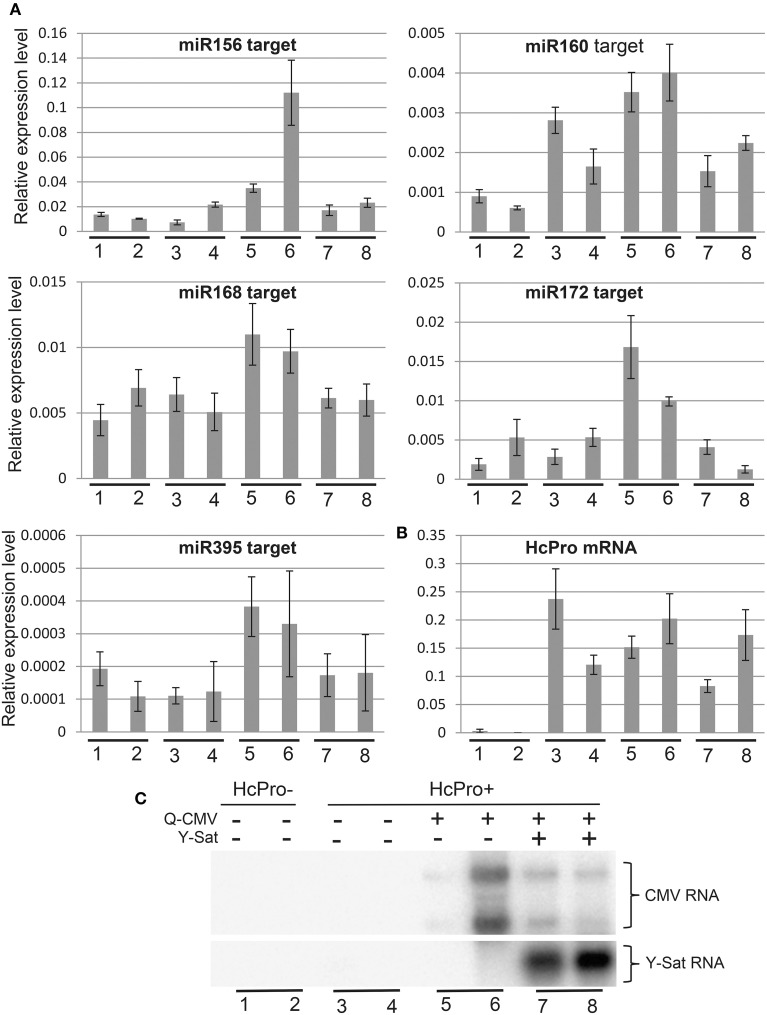
**The increased expression of miRNA target genes by CMV infection in HcPro-expressing *N. tabacum* plants is reversed in the presence Y-Sat**. **(A)** qRT-PCR analysis of predicted target genes for miR156, miR160, miR168, miR172, and miR395. The EF1α gene was used as the internal reference. **(B)** qRT-PCR analysis of HcPro expression in the HcPro plants. **(C)** Northern blot analysis of Q-CMV and Y-Sat levels in the transgenic HcPro *N. tabacum* plants used for qRT-PCR analysis. HcPro+, *N. tabacum* plants containing the HcPro transgene; HcPro-, *N. tabacum* segregants containing no HcPro transgene.

VSRs, including CMV 2b, suppress the function of both viral and host siRNAs and miRNAs (Brigneti et al., 1998; Kasschau et al., 2003; Pumplin and Voinnet, 2013). Our agro-infiltration assays indicated that Y-Sat infection interferes with the VSR effect on siRNA-directed gene silencing in the host plant. To determine if Y-Sat infection affected the function of VSRs on host miRNA-directed gene silencing, expression levels of target genes of five miRNAs (miR156, miR160, miR168, miR172, and miR395) in the Q-CMV and Q-CMV+Y-Sat-infected transgenic *N. tabacum* plants overexpressing potyvirus (*Tobacco etch virus*)-encoded VSR HcPro were analyzed. The HcPro plants rather than wild-type plants were used for two reasons. First, the plants do not develop yellowing symptoms with Q-CMV+Y-Sat infection, presumably because Y-Sat siRNA-directed silencing of the chlorophyll biosynthetic gene *CHLI* is repressed by HcPro (Shimura et al., 2011; Smith et al., 2011; Shen et al., 2014). This minimizes the possible effect of the yellowing symptoms on the expression of miRNAs' targets. Second, infection of the HcPro plants, but not wild-type plants, with Q-CMV resulted in consistent upregulation of miRNA target gene expression (Figure 4A and data not shown), possibly because of a synergistic effect between CMV 2b and HcPro or the relatively high level of CMV accumulation in HcPro plants in comparison to wild-type plants. This allowed us to examine the effect of Y-Sat on VSR-mediated mis-regulation of miRNA target genes. It is worth noting that HcPro increases the expression levels of several miRNAs' targets in *Arabidopsis* (Kasschau et al., 2003), but among the five *N. tabacum* miRNA target genes examined, only that of miR160 appeared to be upregulated in the HcPro-expressing *N. tabacum* plants without CMV infection (Figure 4A). The target genes of the remaining four miRNAs were expressed at similar levels between HcPro-expressing (HcPro+) (samples 3–4) and wild-type (HcPro-) (samples 1–2) *N. tabacum* plants (Figure 4A).

All five miRNA target genes showed increased expression in the CMV-infected HcPro+ plants compared to the uninfected HcPro+ plants (Figure 4A, samples 5–6 vs. 3–4). This suggests that the 2b protein from the infecting CMV, probably in conjunction with the transgene-expressed HcPro, suppressed miRNA-directed silencing resulting in upregulation of the miRNA target genes in the CMV-infected plants. However, in the plants co-infected with CMV and Y-Sat, the expression levels of these target genes were reduced and became similar to those in the uninfected HcPro+ plants (compare samples 7–8 with samples 5–6 or 3–4). This change was not due to a change in HcPro expression because its expression level was similar between the CMV and CMV+Y-Sat-infected plants and showed no correlation with the expression of miRNA target genes (Figure 4B). Furthermore, this change was not caused by a change in CMV RNA level because there was in general no correlation between the expression level of miRNA target genes and the amount of CMV RNA in the CMV and CMV+Y-Sat-infected plants (Figures 4A,C). The expression of the miR156 target gene in the two CMV-infected plants appeared to be an exception, which showed a positive correlation with the amount of CMV RNA (Figures 4A,C). Taken together, the decrease in miRNA target gene expression upon Y-Sat infection suggests that the suppression of miRNA function by CMV 2b in conjunction with HcPro was overcome by Y-Sat.

### Y-Sat siRNAs bind to P19 and sequester P19 from *hpGUS*-derived siRNAs

**Figure 5:**
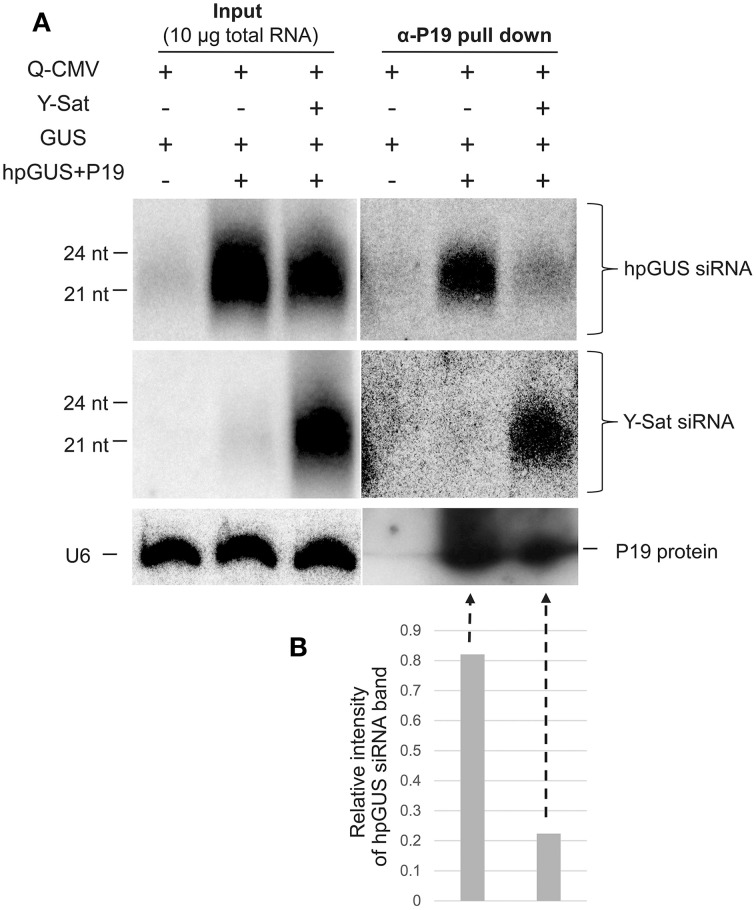
**RNA immunoprecipitation assay shows reduced binding of *hpGUS*-derived siRNAs to P19 in the presence of Y-Sat**. **(A)** The amounts of GUS and Y-Sat siRNAs from the input (left three lanes) and RNA-IP (right three lanes) were determined by northern blot hybridization, which showed a strong reduction of hpGUS-derived siRNAs (top right panel) and abundant Y-Sat-derived siRNAs (middle right panel) in the presence of Y-Sat. The amounts of P19 protein in the RNA-IP pull-down product are indicated by Western blot (bottom right panel). The sizes of hpGUS- and Y-Sat-derived siRNAs are indicated. U6 small nuclear RNA hybridized by the U6 probe described above was used as a loading control for the input samples (bottom left panel). **(B)** Relative intensity of the α-P19 pull-down hpGUS siRNA band (the last two lanes of the top panel) after normalization against the P19 protein band (the last two lanes of the bottom panel).

As shown in Figure 1, Y-Sat-derived siRNAs were highly abundant in the infected plants. This led us to test if the satRNA-derived siRNAs (sat-siRNAs) might bind and sequester the VSRs. RNA immunoprecipiation (RNA-IP) was performed on the *N. benthamiana* tissues agro-infiltrated with *GUS*+*hpGUS*+*P19* using the P19 antibody (α-P19). Northern analysis of the P19-bound sRNAs showed that Y-Sat-siRNAs were present at high levels in the α-P19 RNA-IP from the Q-CMV+Y-Sat-infected tissues (Figure 5A, middle-right panel). In contrast, the amount of *hpGUS*-derived siRNAs in the α-P19 RNA-IP was dramatically reduced compared to that in the plants infected by Q-CMV alone without Y-Sat (Figure 5A, top-right panel and Figure 5B). These results indicate that P19 suppresses the silencing of *GUS* expression via binding to the *hpGUS*-derived siRNAs, and that in the presence of Y-Sat, the abundant Y-Sat-derived siRNAs bind to P19 reduce its capacity to bind *hpGUS*-derived siRNAs. As a result, Y-Sat infection allowed more *hpGUS*-derived siRNAs to be available for directing GUS silencing. We did not pursue an RNA-IP assay for the binding of Y-Sat siRNAs to VSR 2b due to the unavailability of 2b antibody. However, from the previous studies showing that 2b also suppresses RNA silencing by binding sRNAs (Vargason et al., 2003; Baulcombe and Molnar, 2004; Gonzalez et al., 2012), it is expected that the abundant Y-Sat-derived siRNAs would bind to 2b and reduces the amount of 2b-bound *hpGUS*-derived siRNAs, enhancing GUS silencing in the *N. benthamiana* tissues agro-infiltrated with *GUS*+*hpGUS*+*2b*.

## Discussion

A previous study on defective interfering RNAs (DI-RNAs) associated with Tomato bushy stunt tombusvirus (TBSV) has shown that abundant siRNAs derived from the DI-RNAs can saturate TBSV-encoded P19 VSR and elevate the levels of free TBSV-derived siRNAs (Havelda et al., 2005). These free TBSV-derived siRNAs in the presence of the DI-RNAs have been proposed to play a role in directing silencing against the helper virus, thereby reducing TBSV accumulation and symptom severity (Havelda et al., 2005). A recent study on a CMV satRNA has also implicated increased anti-helper virus RNA silencing and reduced helper virus accumulation in satRNA-mediated symptom attenuation (Hou et al., 2011). This study shows that satRNA reduces the accumulation of VSR 2b. Because 2b interferes with the function of viral sRNAs, it is likely that satRNA enhances anti-viral silencing (Hou et al., 2011) which in turn reduces the accumulation of CMV helper virus.

Whereas increased antiviral silencing and reduced helper virus accumulation as proposed in the two studies may well play a role in satRNA-mediated symptom attenuation, our results suggest an alternative mechanism by which satRNAs reduce helper virus-caused symptoms. Similar to the DI RNA scenario, satRNA-derived siRNAs bind to and saturate VSRs due to their high abundance in virus-infected tissues. However, in addition to promoting RNA silencing against the helper virus to reduce its accumulation, this minimizes the capacity of VSRs to bind host endogenous siRNAs and miRNAs. As a result, these endogenous siRNAs and miRNAs are free to form RISC with AGO proteins allowing normal sRNA-mediated gene regulation to occur in the host plants. Thus, developmental abnormalities, or symptoms, caused by the helper virus VSRs are minimized in the presence of satRNAs.

We have demonstrated that Y-Sat effectively reduced the ability of 2b and P19 in suppressing the hpRNA-induced GUS silencing in *N. benthamiana* plants using the agro-infiltration-based transient expression assay. We have also shown that CMV infection increased the expression levels of miRNAs' target genes but in the presence of Y-Sat the expression levels of these target genes reverted to uninfected levels. Taken together, our data indicate that Y-Sat can impact on both the siRNA- and miRNA-directed RNA silencing pathways in the host plants. CMV 2b was previously shown to bind both siRNAs and AGO proteins (Zhang et al., 2006; Duan et al., 2012; Hamera et al., 2012). However, *in vivo* suppression of RNA silencing requires its siRNA binding activity but is independent of its ability to interact directly with AGOs (Duan et al., 2012). We thus expect that binding and saturation of the 2b VSR by sat-siRNAs minimizes the ability of 2b to bind host sRNAs and suppress host sRNA-directed RNA silencing, which would restore host sRNA-mediated gene regulation that would benefit normal plant development resulting in attenuated viral symptoms.

It is interesting to note that CMV infection alone seemed to affect the function of the ago-infiltrated 2b and P19 VSRs. There is a reduction in GUS expression in the CMV-infected in comparison to uninfected plants in *GUS*+*hpGUS*+*2b*-infiltrated (Figure 2A) as well as GUS+*hpGUS*+*P19*-infiltrated (Figure 3A) tissues. A possible explanation is that CMV-derived siRNAs, although less abundant than Y-Sat-derived siRNAs, also bind to the VSRs reducing their capacity to suppress hpRNA-directed GUS silencing. This result could imply that viral siRNAs not only play a role in directing silencing against the viruses, but also contribute to the minimization of VSR's interference with host plant sRNA functions.

satRNA-derived siRNAs have the potential to compete with host plant-encoded sRNAs for endogenous RNA silencing machineries such as DCLs and AGO proteins, which in turn could affect host sRNA-directed gene regulation. Thus, the high abundance of satRNA siRNAs could both reduce symptoms by binding to VSRs and exacerbate symptoms by competing for host silencing machineries. However, we have shown here and previously (Shen et al., 2014) that the high abundance of Y-Sat siRNAs does not seem to affect the biogenesis of host sRNAs in *Nicotiana* plants. Furthermore, in the absence of a strong VSR and visible helper virus-caused symptoms (namely in the case of the infection of *N. benthamiana* with Q-CMV which encodes a weak 2b), host sRNA-directed silencing and plant growth are not affected by the high level of Y-Sat siRNAs (Shen et al., 2014). Why host silencing machineries are not saturated by the abundant satRNA siRNAs remains to be further understood, but these results suggest that satRNA-derived siRNAs affect host sRNA functions mainly through interacting with VSRs.

In conclusion, our results suggest that satRNAs are likely to attenuate the symptoms via their abundant siRNAs, which bind helper virus-encoded VSRs minimizing their interference with the endogenous siRNA and miRNA pathways as well as the antiviral silencing pathways as proposed in the previous studies. This model could apply to other viral or subviral RNAs that give rise to high levels of siRNAs in infected host cells, for example, DI RNAs. DI RNAs are a truncated form of their associated helper viral genomes and, like satRNAs, also depend on the helper virus for replication and can also attenuate helper virus-induced symptoms (Simon et al., 2004). Unlike satRNAs that occur mostly in plants, DI RNAs are widespread and associated with both plant viruses and animal viruses. Because animal viruses also encode VSRs such as the B2 protein encoded by Flock House virus (FHV) (Aliyari et al., 2008), it would be valuable to examine if DI RNAs modulate animal viral symptoms through a similar mechanism. If so, potential DI RNA-related strategies could be developed against animal viral diseases in the future.

### Conflict of interest statement

The authors declare that the research was conducted in the absence of any commercial or financial relationships that could be construed as a potential conflict of interest.
