# Supplementary material for: Satellite RNAs interfere with the function of viral RNA silencing suppressors
Source: Front Plant Sci. 2015 Apr 24;6:281. doi: 10.3389/fpls.2015.00281 (PMC4408847; doi:10.3389/fpls.2015.00281)
Supplement: Supplementary file 1 [file Presentation1.PPTX]

## Slide 1
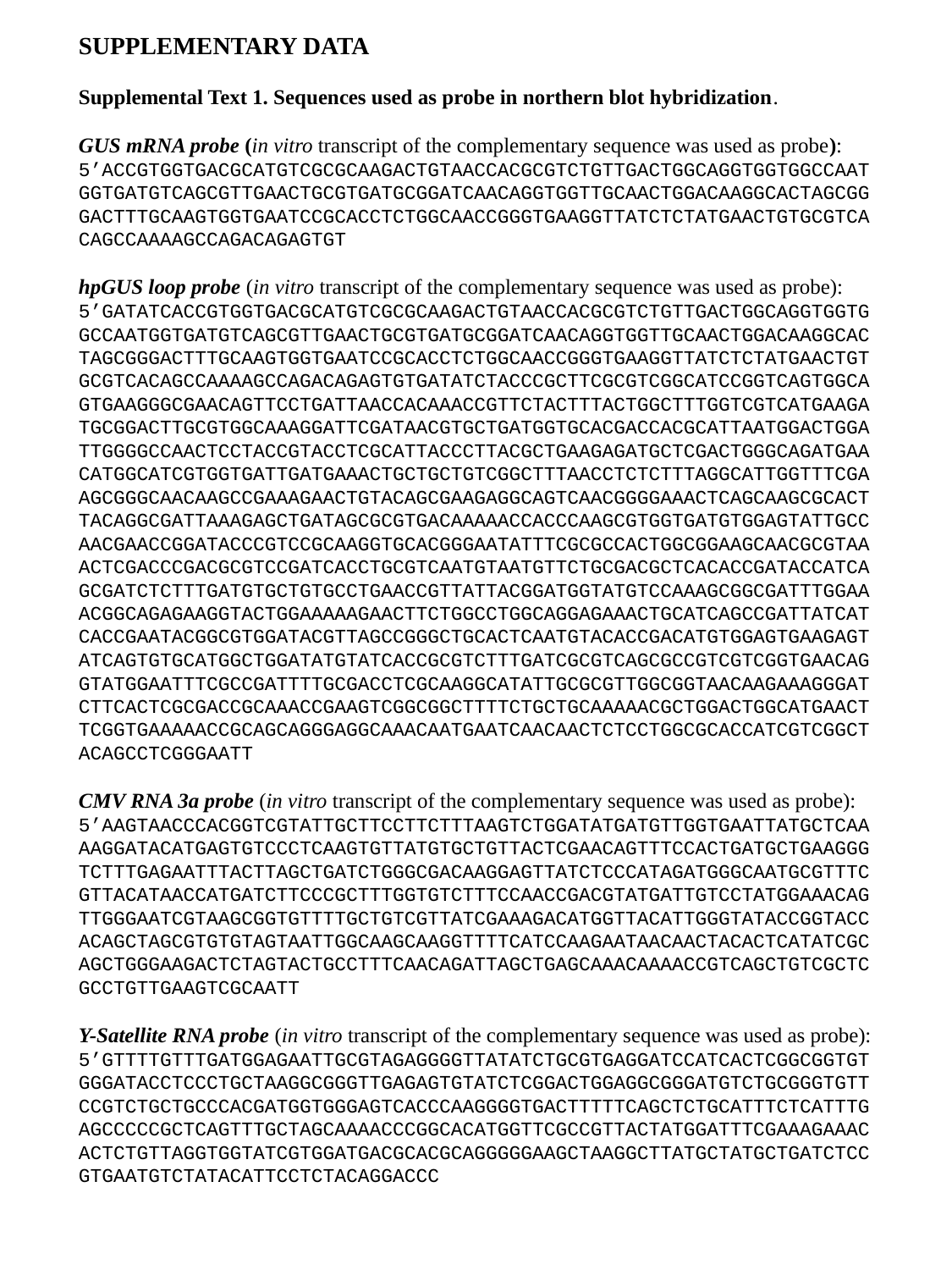

SUPPLEMENTARY DATA
Supplemental Text 1. Sequences used as probe in northern blot hybridization.
GUS mRNA probe (in vitro transcript of the complementary sequence was used as probe):
5’ACCGTGGTGACGCATGTCGCGCAAGACTGTAACCACGCGTCTGTTGACTGGCAGGTGGTGGCCAATGGTGATGTCAGCGTTGAACTGCGTGATGCGGATCAACAGGTGGTTGCAACTGGACAAGGCACTAGCGGGACTTTGCAAGTGGTGAATCCGCACCTCTGGCAACCGGGTGAAGGTTATCTCTATGAACTGTGCGTCACAGCCAAAAGCCAGACAGAGTGT
hpGUS loop probe (in vitro transcript of the complementary sequence was used as probe):
5’GATATCACCGTGGTGACGCATGTCGCGCAAGACTGTAACCACGCGTCTGTTGACTGGCAGGTGGTGGCCAATGGTGATGTCAGCGTTGAACTGCGTGATGCGGATCAACAGGTGGTTGCAACTGGACAAGGCACTAGCGGGACTTTGCAAGTGGTGAATCCGCACCTCTGGCAACCGGGTGAAGGTTATCTCTATGAACTGTGCGTCACAGCCAAAAGCCAGACAGAGTGTGATATCTACCCGCTTCGCGTCGGCATCCGGTCAGTGGCAGTGAAGGGCGAACAGTTCCTGATTAACCACAAACCGTTCTACTTTACTGGCTTTGGTCGTCATGAAGATGCGGACTTGCGTGGCAAAGGATTCGATAACGTGCTGATGGTGCACGACCACGCATTAATGGACTGGATTGGGGCCAACTCCTACCGTACCTCGCATTACCCTTACGCTGAAGAGATGCTCGACTGGGCAGATGAACATGGCATCGTGGTGATTGATGAAACTGCTGCTGTCGGCTTTAACCTCTCTTTAGGCATTGGTTTCGAAGCGGGCAACAAGCCGAAAGAACTGTACAGCGAAGAGGCAGTCAACGGGGAAACTCAGCAAGCGCACTTACAGGCGATTAAAGAGCTGATAGCGCGTGACAAAAACCACCCAAGCGTGGTGATGTGGAGTATTGCCAACGAACCGGATACCCGTCCGCAAGGTGCACGGGAATATTTCGCGCCACTGGCGGAAGCAACGCGTAAACTCGACCCGACGCGTCCGATCACCTGCGTCAATGTAATGTTCTGCGACGCTCACACCGATACCATCAGCGATCTCTTTGATGTGCTGTGCCTGAACCGTTATTACGGATGGTATGTCCAAAGCGGCGATTTGGAAACGGCAGAGAAGGTACTGGAAAAAGAACTTCTGGCCTGGCAGGAGAAACTGCATCAGCCGATTATCATCACCGAATACGGCGTGGATACGTTAGCCGGGCTGCACTCAATGTACACCGACATGTGGAGTGAAGAGTATCAGTGTGCATGGCTGGATATGTATCACCGCGTCTTTGATCGCGTCAGCGCCGTCGTCGGTGAACAGGTATGGAATTTCGCCGATTTTGCGACCTCGCAAGGCATATTGCGCGTTGGCGGTAACAAGAAAGGGATCTTCACTCGCGACCGCAAACCGAAGTCGGCGGCTTTTCTGCTGCAAAAACGCTGGACTGGCATGAACTTCGGTGAAAAACCGCAGCAGGGAGGCAAACAATGAATCAACAACTCTCCTGGCGCACCATCGTCGGCTACAGCCTCGGGAATT
CMV RNA 3a probe (in vitro transcript of the complementary sequence was used as probe):
5’AAGTAACCCACGGTCGTATTGCTTCCTTCTTTAAGTCTGGATATGATGTTGGTGAATTATGCTCAAAAGGATACATGAGTGTCCCTCAAGTGTTATGTGCTGTTACTCGAACAGTTTCCACTGATGCTGAAGGGTCTTTGAGAATTTACTTAGCTGATCTGGGCGACAAGGAGTTATCTCCCATAGATGGGCAATGCGTTTCGTTACATAACCATGATCTTCCCGCTTTGGTGTCTTTCCAACCGACGTATGATTGTCCTATGGAAACAGTTGGGAATCGTAAGCGGTGTTTTGCTGTCGTTATCGAAAGACATGGTTACATTGGGTATACCGGTACCACAGCTAGCGTGTGTAGTAATTGGCAAGCAAGGTTTTCATCCAAGAATAACAACTACACTCATATCGCAGCTGGGAAGACTCTAGTACTGCCTTTCAACAGATTAGCTGAGCAAACAAAACCGTCAGCTGTCGCTCGCCTGTTGAAGTCGCAATT
Y-Satellite RNA probe (in vitro transcript of the complementary sequence was used as probe):
5’GTTTTGTTTGATGGAGAATTGCGTAGAGGGGTTATATCTGCGTGAGGATCCATCACTCGGCGGTGTGGGATACCTCCCTGCTAAGGCGGGTTGAGAGTGTATCTCGGACTGGAGGCGGGATGTCTGCGGGTGTTCCGTCTGCTGCCCACGATGGTGGGAGTCACCCAAGGGGTGACTTTTTCAGCTCTGCATTTCTCATTTGAGCCCCCGCTCAGTTTGCTAGCAAAACCCGGCACATGGTTCGCCGTTACTATGGATTTCGAAAGAAACACTCTGTTAGGTGGTATCGTGGATGACGCACGCAGGGGGAAGCTAAGGCTTATGCTATGCTGATCTCCGTGAATGTCTATACATTCCTCTACAGGACCC

## Slide 2
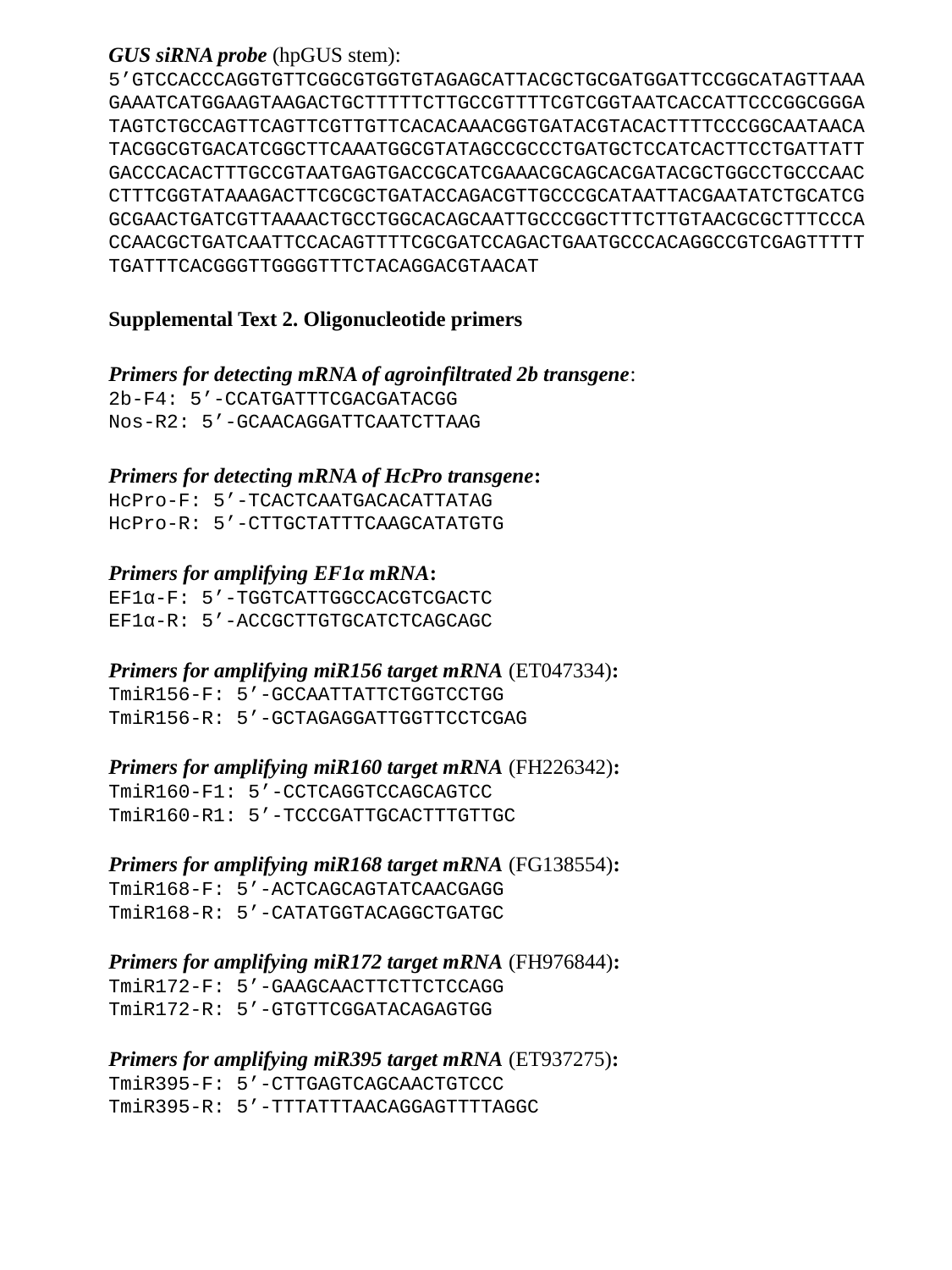

GUS siRNA probe (hpGUS stem):
5’GTCCACCCAGGTGTTCGGCGTGGTGTAGAGCATTACGCTGCGATGGATTCCGGCATAGTTAAAGAAATCATGGAAGTAAGACTGCTTTTTCTTGCCGTTTTCGTCGGTAATCACCATTCCCGGCGGGATAGTCTGCCAGTTCAGTTCGTTGTTCACACAAACGGTGATACGTACACTTTTCCCGGCAATAACATACGGCGTGACATCGGCTTCAAATGGCGTATAGCCGCCCTGATGCTCCATCACTTCCTGATTATTGACCCACACTTTGCCGTAATGAGTGACCGCATCGAAACGCAGCACGATACGCTGGCCTGCCCAACCTTTCGGTATAAAGACTTCGCGCTGATACCAGACGTTGCCCGCATAATTACGAATATCTGCATCGGCGAACTGATCGTTAAAACTGCCTGGCACAGCAATTGCCCGGCTTTCTTGTAACGCGCTTTCCCACCAACGCTGATCAATTCCACAGTTTTCGCGATCCAGACTGAATGCCCACAGGCCGTCGAGTTTTTTGATTTCACGGGTTGGGGTTTCTACAGGACGTAACAT
Supplemental Text 2. Oligonucleotide primers
Primers for detecting mRNA of agroinfiltrated 2b transgene:
2b-F4: 5’-CCATGATTTCGACGATACGG
Nos-R2: 5’-GCAACAGGATTCAATCTTAAG
Primers for detecting mRNA of HcPro transgene:
HcPro-F: 5’-TCACTCAATGACACATTATAG
HcPro-R: 5’-CTTGCTATTTCAAGCATATGTG
Primers for amplifying EF1α mRNA:
EF1α-F: 5’-TGGTCATTGGCCACGTCGACTC
EF1α-R: 5’-ACCGCTTGTGCATCTCAGCAGC
Primers for amplifying miR156 target mRNA (ET047334):
TmiR156-F: 5’-GCCAATTATTCTGGTCCTGG
TmiR156-R: 5’-GCTAGAGGATTGGTTCCTCGAG
Primers for amplifying miR160 target mRNA (FH226342):
TmiR160-F1: 5’-CCTCAGGTCCAGCAGTCC
TmiR160-R1: 5’-TCCCGATTGCACTTTGTTGC
Primers for amplifying miR168 target mRNA (FG138554):
TmiR168-F: 5’-ACTCAGCAGTATCAACGAGG
TmiR168-R: 5’-CATATGGTACAGGCTGATGC
Primers for amplifying miR172 target mRNA (FH976844):
TmiR172-F: 5’-GAAGCAACTTCTTCTCCAGG
TmiR172-R: 5’-GTGTTCGGATACAGAGTGG
Primers for amplifying miR395 target mRNA (ET937275):
TmiR395-F: 5’-CTTGAGTCAGCAACTGTCCC
TmiR395-R: 5’-TTTATTTAACAGGAGTTTTAGGC

## Slide 3
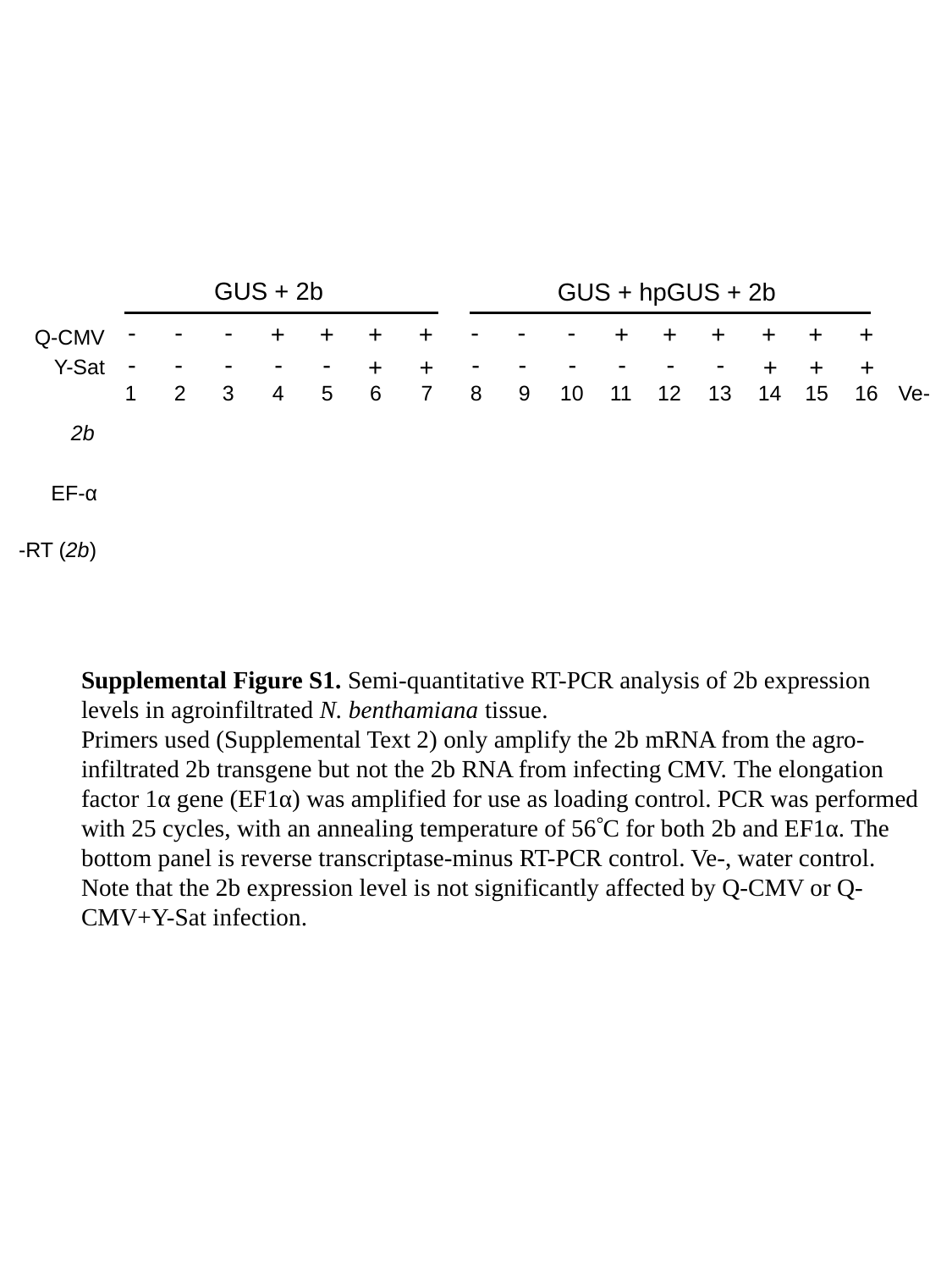

GUS + 2b
GUS + hpGUS + 2b
-
-
-
-
-
-
+
+
+
+
+
+
+
+
+
+
Q-CMV
-
-
-
-
-
-
-
-
-
-
-
+
+
+
+
+
Y-Sat
Ve-
13
16
6
9
10
12
15
3
5
8
11
14
2
4
7
1
2b
EF-α
-RT (2b)
Supplemental Figure S1. Semi-quantitative RT-PCR analysis of 2b expression levels in agroinfiltrated N. benthamiana tissue.
Primers used (Supplemental Text 2) only amplify the 2b mRNA from the agro-infiltrated 2b transgene but not the 2b RNA from infecting CMV. The elongation factor 1α gene (EF1α) was amplified for use as loading control. PCR was performed with 25 cycles, with an annealing temperature of 56°C for both 2b and EF1α. The bottom panel is reverse transcriptase-minus RT-PCR control. Ve-, water control. Note that the 2b expression level is not significantly affected by Q-CMV or Q-CMV+Y-Sat infection.
